# DNA polymerase θ promotes CAG•CTG repeat expansions in Huntington’s disease *via* insertion sequences of its catalytic domain

**DOI:** 10.1016/j.jbc.2021.101144

**Published:** 2021-08-30

**Authors:** Kara Y. Chan, Xueying Li, Janice Ortega, Liya Gu, Guo-Min Li

**Affiliations:** 1 Department of Radiation Oncology, University of Texas Southwestern Medical Center, Dallas, Texas, USA; 2 Department of Toxicology and Cancer Biology, University of Kentucky College of Medicine, Lexington, Kentucky, USA

**Keywords:** DNA polymerase θ, triplet repeats, DNA hairpin, Huntington’s disease, 8-oxoG, 8-oxo-guanine, AP, abasic, BER, base excision repair, FBS, fetal bovine serum, HD, Huntington’s disease, HEK, human embryonic kidney, OGG1, 8-oxo-guanine DNA glycosylase 1, PCNA, proliferating cellular nuclear antigen, Polβ, polymerase β, Polθ, DNA polymerase θ, PolθΔi2, insertion 2–depleted Polθ, RFC, replication factor C, ROS, reactive oxygen species, TdT, terminal deoxynucleotidyl transferase, TNRs, trinucleotide repeats, WCLs, whole-cell lysates

## Abstract

Huntington's disease (HD), a neurodegenerative disease characterized by progressive dementia, psychiatric problems, and chorea, is known to be caused by CAG repeat expansions in the HD gene *HTT*. However, the mechanism of this pathology is not fully understood. The translesion DNA polymerase θ (Polθ) carries a large insertion sequence in its catalytic domain, which has been shown to allow DNA loop-outs in the primer strand. As a result of high levels of oxidative DNA damage in neural cells and Polθ's subsequent involvement in base excision repair of oxidative DNA damage, we hypothesized that Polθ contributes to CAG repeat expansion while repairing oxidative damage within *HTT*. Here, we performed Polθ-catalyzed *in vitro* DNA synthesis using various CAG•CTG repeat DNA substrates that are similar to base excision repair intermediates. We show that Polθ efficiently extends (CAG)_n_•(CTG)_n_ hairpin primers, resulting in hairpin retention and repeat expansion. Polθ also triggers repeat expansions to pass the threshold for HD when the DNA template contains 35 repeats upward. Strikingly, Polθ depleted of the catalytic insertion fails to induce repeat expansions regardless of primers and templates used, indicating that the insertion sequence is responsible for Polθ's error-causing activity. In addition, the level of chromatin-bound Polθ in HD cells is significantly higher than in non-HD cells and exactly correlates with the degree of CAG repeat expansion, implying Polθ's involvement in triplet repeat instability. Therefore, we have identified Polθ as a potent factor that promotes CAG•CTG repeat expansions in HD and other neurodegenerative disorders.

The expansion of trinucleotide repeats (TNRs) is associated with a number of neurodegenerative disorders, including Huntington’s disease (HD) and myotonic dystrophy (1, 2). Once the expansion exceeds a certain threshold, for example, 35 CAG repeats in HD, it inactivates the expression and/or alters the function of the affected genes, which leads to disease onset. Despite extensive studies, how the TNR expands is still not fully understood. Elucidating the mechanisms of TNR expansion will significantly impact the therapeutic approaches for diseases caused by this process (3, 4).

Several DNA metabolic pathways, including DNA replication and repair, have been implicated in TNR instability (1, 2, 5, 6, 7, 8, 9, 10). A common feature associated with all DNA metabolic reactions is DNA breaks or free DNA ends, which can induce the formation of DNA hairpins within TNRs *via* strand slippage (5, 11, 12). DNA hairpin formation results in TNR expansion (1, 2, 10, 11, 12) when these hairpins are not removed (13, 14). Because cell division does not occur in the human brain, CAG/CTG repeat expansions in HD and other neurodegenerative disorders may not be related to DNA replication, but to DNA repair.

Reactive oxygen species (ROS) are a major source of DNA damage in the human brain, and guanine, which is enriched in CAG/CTG repeats, is the favored target of ROS (15). The repair of oxidative adduct 8-oxo-guanine (8-oxoG) by the 8-oxo-guanine DNA glycosylase 1 (OGG1) has been implicated in CAG/CTG repeat expansions, as depleting *Ogg1* abolishes age-dependent CAG repeat expansion in HD mouse models (16). OGG1-initiated base excision repair (OGG1-BER) involves lesion recognition, flipping the lesion from the DNA double helix into the base-binding pocket (active site) of OGG1, and site-specific changes in the DNA structure (17, 18). As a bifunctional DNA glycosylase, OGG1 excises the oxidized guanine base to generate an abasic (AP) site by using its glycosylase activity and cleaves the phosphodiester bond 3′ to the AP site by using its AP lyase activity. AP endonuclease 1 cuts the phosphodiester backbone immediately 5′ to the AP site. These cleavages result in a one-nucleotide gap, which is filled in by DNA polymerase β (Polβ), followed by DNA ligase III–catalyzed ligation (19). However, the OGG1 AP lyase activity is ∼500-fold less efficient than its glycosylase activity (20). In this case, cleavage of the AP site by AP endonuclease 1 leaves a 3′-hydroxyl and 5′-deoxyribose phosphate (5′-dRP) terminus. The latter can be removed by Polβ's 5′-dRP lyase activity (21). Alternatively, flap-endonuclease 1, which is required for long-patch BER, can remove the 5′-dRP to produce an ssDNA gap with 1 to 4 nucleotides, depending on the DNA polymerase involved in long-patch BER (19). Regardless, the ssDNA gap generated by BER promotes hairpin formation within CAG repeats *via* strand slippage (16, 22). Subsequently, Polβ fills the DNA gap by efficiently utilizing a CAG hairpin structure as a primer for DNA synthesis, which leads to CAG repeat expansion (23). Consistently, there is a close association between age-dependent somatic CAG repeat expansion and oxidative DNA damage in HD mouse models (24, 25), and both OGG1 and Polβ have been shown to promote CAG repeat expansions (16, 22, 23, 26, 27).

In addition to Polβ, DNA polymerase θ (Polθ), a low-fidelity family A DNA polymerase (28), participates in BER of oxidative DNA damage (29). Polθ is known to add single nucleotides to homopolymeric runs at a high rate (30). This is probably related to Polθ's large insertion loops (Fig. 1*A*), particularly insertion 2 in the polymerase thumb domain (31). Recent structural studies have revealed that the presence of insertion 2 creates a big flexible cavity in the DNA-binding surface (32, 33). Strikingly, Polθ can extend a loop-out–containing primer (32), an activity also associated with Polβ (34). Therefore, we hypothesized that Polθ is a major polymerase that promotes CAG/CTG repeat expansions during DNA synthesis.

**Figure 1:**
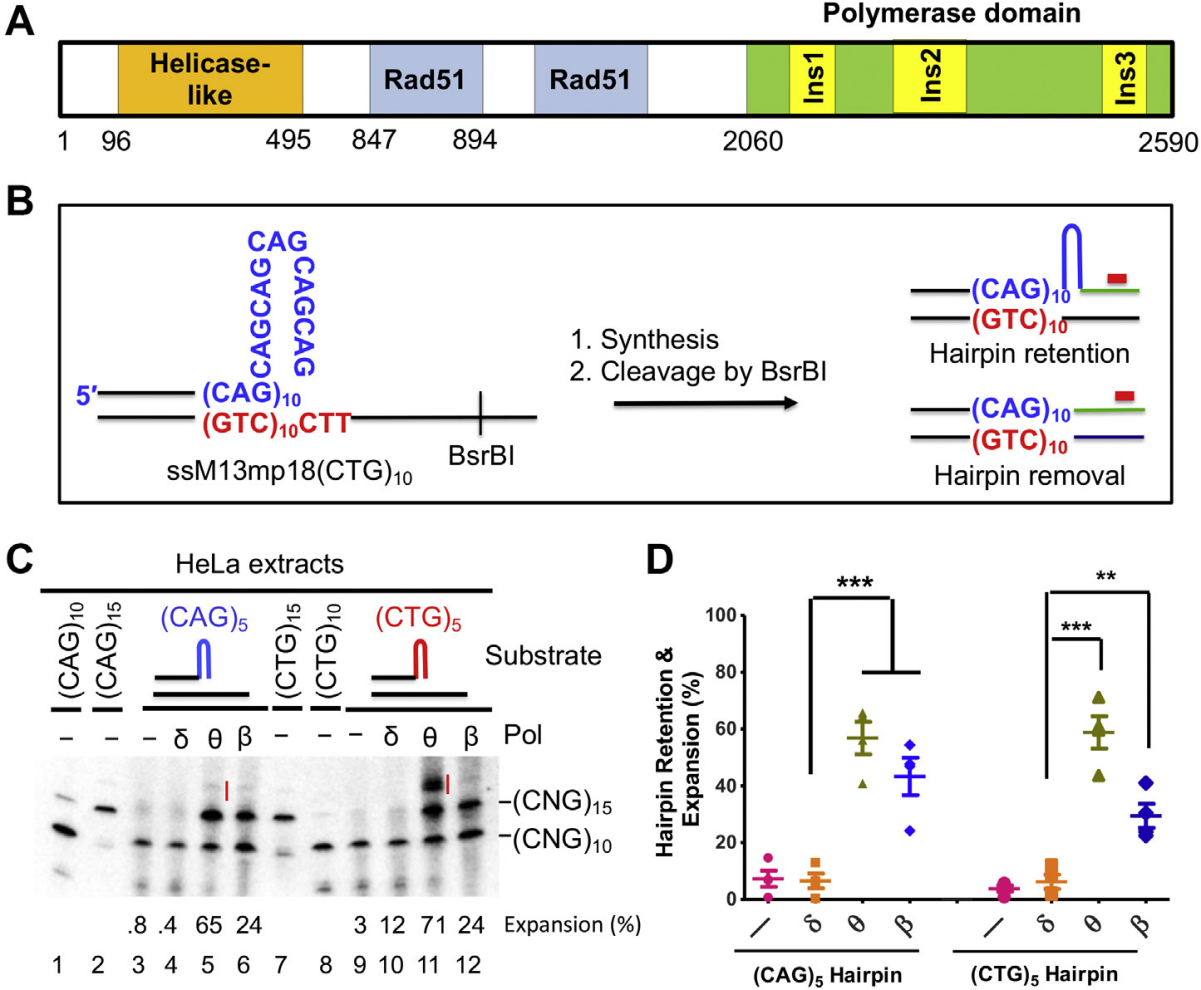
**Polθ promotes CAG/CTG repeat expansion.***A*, diagram of Polθ functional domains. The polymerase domain contains three insertion loop motifs. *B*, illustration of hairpin primer extension-Southern hybridization assay. *Red bars* represent ^32^P-labeled probe. *C*, hairpin primer extension assay shows Polθ's ability to use a CAG or CTG hairpin as a primer for DNA synthesis, leading to hairpin retention and repeat expansions beyond the hairpin. *D*, quantification and comparison of hairpin retention and repeat expansions by individual polymerases, as indicated. Data are from four independent determinations. ∗∗∗*p* < 0.001. Polθ, DNA polymerase θ; PolθΔi2, insertion 2–depleted Polθ.

We performed Polθ-catalyzed DNA synthesis using a series of BER intermediates containing various numbers of CAG/CTG repeats with or without a hairpin. Like Polβ, Polθ can effectively extend hairpin primers to stabilize hairpin structures, and it induces large expansions when copying a DNA template that contains CAG/CTG repeats. However, a Polθ mutant that lacks insertion 2 does not induce CAG/CTG repeat expansions regardless of the primers and templates used, which suggests that insertion 2 is responsible for Polθ’s error-prone activity. We also found that Polθ’s chromatin level is significantly higher in HD cells than in non-HD cells and closely correlates with the degree of CAG repeat expansion, which implies the involvement of Polθ in CAG repeat expansion. Therefore, this study has identified Polθ as a potent factor that promotes the CAG/CTG repeat expansions that cause HD and other neurodegenerative diseases.

## Results

### Polθ extends CAG/CTG hairpin primers *in vitro*

To determine whether Polθ can extend a CAG or CTG hairpin primer, we conducted *in vitro* DNA synthesis using a limited amount of HeLa nuclear extracts supplemented with a purified 90-kD catalytic Polθ polypeptide (Fig. S1) (32), a (CAG)_5_ or (CTG)_5_ hairpin primer that anneals to ssM13mp18(CTG)_10_ or ssM13mp18(CAG)_10_ (Fig. 1*B*), respectively, as described previously (23). The reaction products were fractionated by denaturing PAGE, followed by Southern blot analysis using a probe that specifically recognizes the downstream sequence near the BsrBI site of the newly synthesized strand (Fig. 1*B*, red bar). Thus, whether the (CAG)_5_ or (CTG)_5_ hairpin is removed or retained can be readily determined based on its mobility during gel electrophoresis (Fig. 1*B*).

Consistent with our previous observations (23), incubating the (CAG)_5_ hairpin substrate with HeLa nuclear extracts supplemented with the proofreading-active Polδ, which removes the hairpin structure, generated a major product that migrates similarly to the (CAG)_10_-containing DNA fragment (Fig. 1*C*, lane 4). Supplementing the reaction with Polβ yielded two major products: a hairpin-retained (CAG)_15_ product and a hairpin-removed (CAG)_10_ product (Fig. 1*C*, lane 6). When purified Polθ replaced Polβ or Polδ in the synthesis reaction, most yielded products were the same as those observed in the Polβ reaction (Fig. 1*C*, lane 5). This suggests that, like Polβ, Polθ can effectively use a CAG hairpin as a primer for DNA synthesis. In addition, a slowly migrated minor product (product I) was also observed in both the Polθ- and the Polβ-containing reactions (Fig. 1*C*, lanes 5 and 6), which indicates that both polymerases promote CAG repeat expansions beyond the (CAG)_5_ hairpin size.

We then tested Polθ's ability to extend a (CTG)_5_ hairpin primer by using the same *in vitro* DNA synthesis assay. The results revealed that, in addition to the hairpin-retained product, that is, the band migrating at the same place as the (CTG)_15_ band, a slowly migrated product merged as the major one, whose size is similar to that of product I generated by Polβ and Polθ during (CAG)_5_-primed DNA synthesis. This product is much more abundant in the Polθ-catalyzed reaction than in the Polβ-catalyzed one (compare product I between lanes 11 and 12), which suggests that Polθ is more error-prone than Polβ when synthesizing (CTG)_n_-primed DNA. Quantitative analysis revealed that the expanded species (hairpin retained and expanded) account for more than 60% of the synthesized products (Fig. 1, *C* and *D*). Taken together, these data suggest that Polθ can effectively synthesize hairpin-primed DNA to promote CAG/CTG repeat expansions.

### Polθ's insertion 2 is responsible for CAG/CTG repeat expansion

**Figure 2:**
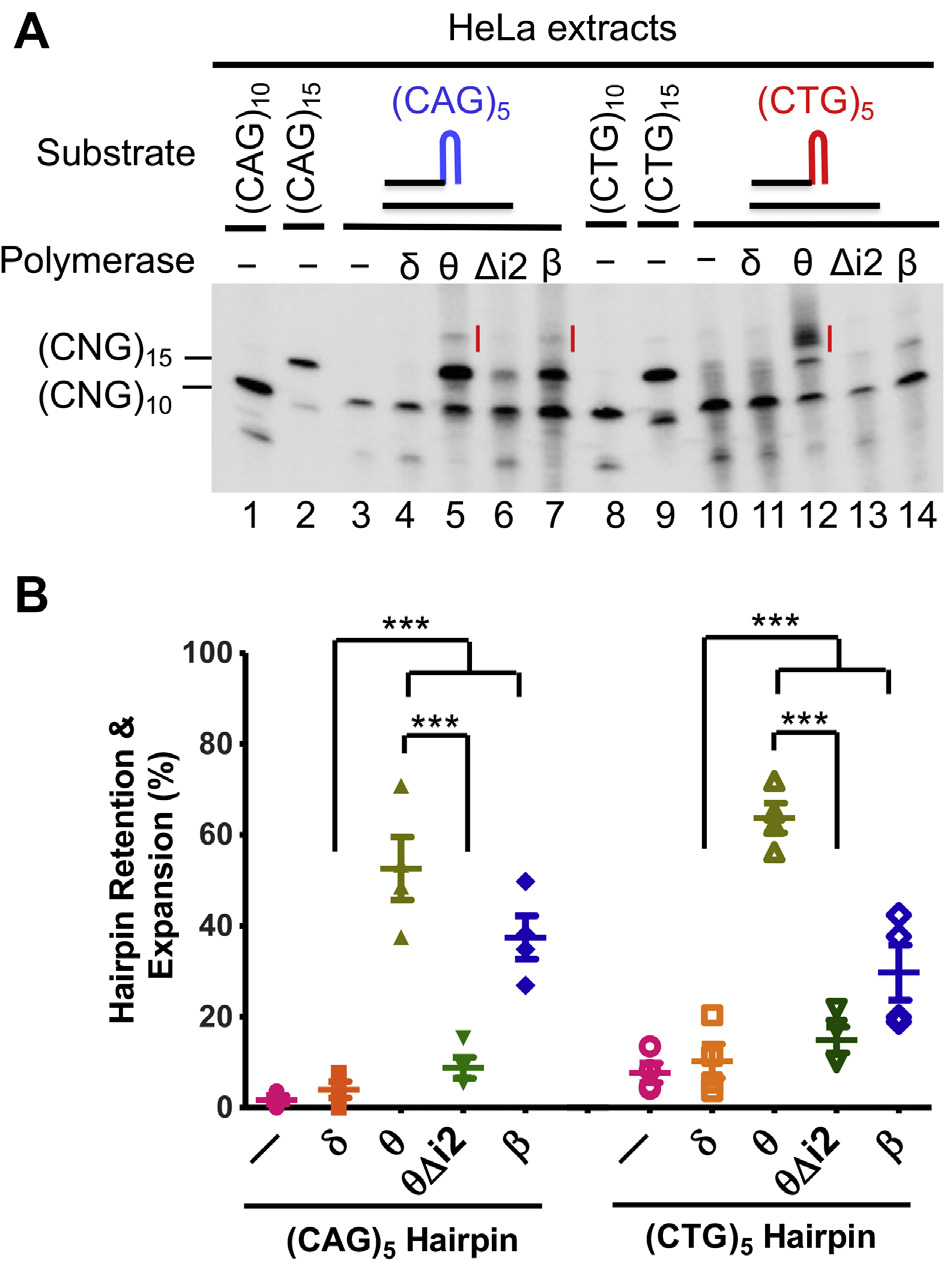
**Insertion 2 of Polθ is responsible for error-prone DNA synthesis.***A*, hairpin primer extension assay showing less error-prone DNA synthesis with PolθΔi2 than with Polθ. *B*, quantification and comparison of error-prone DNA synthesis by individual DNA polymerases, as indicated. Data are from four independent determinations. ∗∗∗*p* < 0.001. Polθ, DNA polymerase θ; PolθΔi2, insertion 2–depleted Polθ.

Polθ's large insertion 2 is responsible for the polymerase’s error-prone nature during DNA synthesis (30, 32, 35). To explore the impact of insertion 2 on the CAG/CTG expansion by Polθ that we observed, we generated an insertion 2–deleted Polθ mutant, as described (35). We examined the mutant Polθ for its ability to synthesize (CAG)_5_- or (CTG)_5_-primed DNA. Remarkably, no product I was generated by the insertion 2–depleted Polθ (PolθΔi2), regardless of whether a (CAG)_5_ or (CTG)_5_ hairpin primer was used (Fig. 2*A*, lanes 6 and 13). In addition, the amount of the hairpin-retained product, that is, (CAG)_15_ or (CTG)_15_, was much lower in the reactions that contained PolθΔi2 (Fig. 2*A*). PolθΔi2 only generated 8.9% of the (CAG)_5_ hairpin–retained and 14.9% of (CTG)_5_ hairpin–retained products produced by Polθ (Fig. 2*A*, compare lane 5 with lane 6, and lane 12 with lane 13). Quantitative analysis shows that this reduction in the hairpin-retained and expanded products in PolθΔi2-catalyzed reactions is highly significant (Fig. 2*B*). These results suggest that insertion 2 is indeed responsible for Polθ-mediated CAG/CTG repeat expansions.

### Polθ-mediated CAG/CTG repeat expansion depends on the repeat length

**Figure 3:**
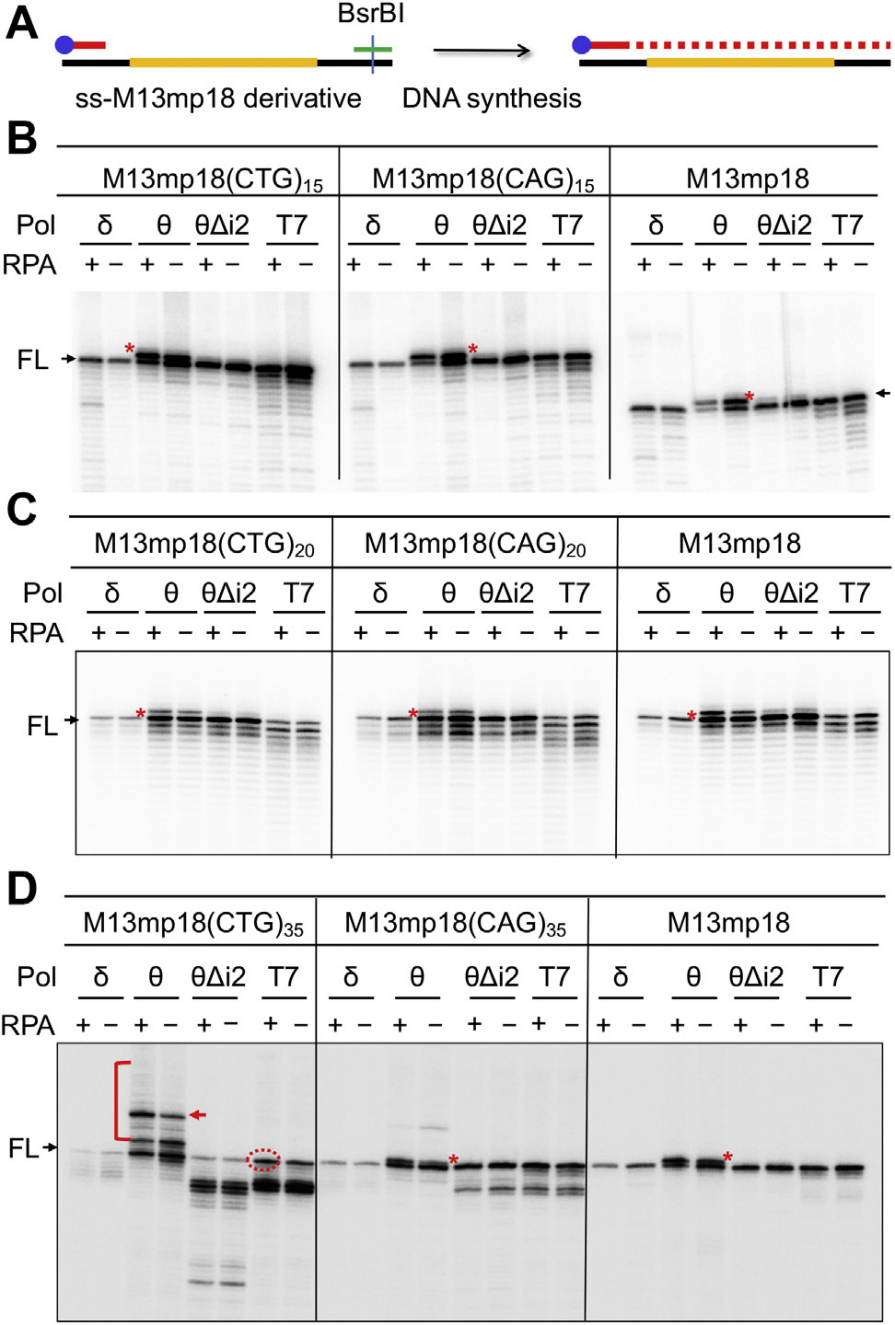
**Polθ's error-prone activity depends on the repeat length.***A*, diagram of *in vitro* DNA synthesis. ssM13mp18 derivatives with various CAG/CTG repeats (*yellow line*) were annealed with an oligonucleotide (*green line*) containing BsrBI restriction recognition sequence and digested with BsrBI before being used as a template for DNA synthesis. *Blue sphere* represents ^32^P-labeling. *B*–*D*, primer extension assays using various CAG and CTG repeat numbers as templates, as indicated. ssDNA of M13mp18 was used as a nonrepeat control. In panel *B*, the same primer was used in all reactions, which is why the products in all M13mp18 reactions are smaller than those in repeat-containing template reactions; in panels *C* and *D*, the primer used in all M13mp18 reactions was adjusted to make the same length products as in repeat-containing template reactions. The DNA bands labeled with a *red arrow* or a *circle* were sequenced. Bands with a *red asterisk* indicate the Polθ TdT-generated products. Polθ, DNA polymerase θ; RPA, replication protein A; TdT, terminal deoxynucleotidyl transferase.

We next examined Polθ's ability to synthesize CAG/CTG repeats by conducting *in vitro* DNA synthesis using a defined DNA synthesis system (Fig. 3*A*) that contains purified Polθ, proliferating cellular nuclear antigen (PCNA) and replication factor C (RFC), a 5′ ^32^P-labeled nonhairpin primer, and M13mp18 derivatives with various lengths of CAG/CTG repeats in the presence or absence of replication protein A, an ssDNA-binding protein that protects ssDNA from nuclease attack and secondary structure formation (36). We observed no expanded DNA products in reactions with high-fidelity polymerases Polδ and T7, regardless of the DNA templates used, that is, (CAG)_20_, (CTG)_20_, (CAG)_25_, (CTG)_25_, (CAG)_35_, (CTG)_35_, or random DNA sequences (Fig. 3, *B*–*D*). PolθΔi2-generated products were essentially the same as those produced by Polδ or T7 polymerase in all reactions (Fig. 3, *B*–*D*), which further suggests that insertion 2 is mutagenic.

However, Polθ-catalyzed products differed from those produced by Polδ, T7, and PolθΔi2. First, in addition to full-length products, Polθ generated a product that is one nucleotide larger than the full-length band in essentially all reactions (see red asterisks in Fig. 3). This one-nucleotide–larger product appears to be unrelated to CAG/CTG repeats, as also seen in reactions with the M13mp18 template. This product is probably derived from Polθ's terminal deoxynucleotidyl transferase (TdT) activity (37), which catalyzes the incorporation of single deoxynucleotides into the 3′-OH terminus of ssDNA or dsDNA. Second, when M13mp18–(CAG)_35_ or M13mp18–(CTG)_35_ was used as a template for DNA synthesis, we detected several expanded products in Polθ-catalyzed reactions (Fig. 3*D*), especially for the M13mp18(CTG)_35_ template (Fig. 3*D*, red bracket). This is probably because a CTG hairpin is easier to form and more stable than a CAG hairpin (11, 23, 38).

To determine the nature of the expansion, we recovered a major Pol-expanded band (Fig. 3*D*, red arrow) and a T7-generated full-length band (Fig. 3*D*, red circle) from the gel. The DNA samples were PCR-amplified, cloned into a vector, and transfected into a bacterial strain. Plasmid DNA samples were isolated from six clones derived from the T7-catalyzed product and 11 clones derived from the Polθ-catalyzed band before DNA-sequencing analysis. The results reveal that in six T7-derived clones, four of them showed original 35 repeats (Fig. S2*A*), and two exhibited 33 repeats, but in 11 Polθ-expanded clones, one clone contained 40 repeats (Fig. S2*B*), seven showed 35 repeats, and three demonstrated 34 repeats. These results are consistent with a previous study, which shows that while the majority of (CTG)_180_ clones undergo contractions after replicating for 100 generations, ∼20% of these clones still remain the full-length repeats (39). We therefore conclude that the 40 repeats represent the original length of the Polθ-expanded band, that is, an expansion of five repeats by Polθ during DNA synthesis (Fig. S2*C*). We did not observe this repeat length–dependent expansion by Polθ in reactions with PolθΔi2 (Fig. 3*D*), which suggests that insertion 2 is also responsible for Polθ-induced repeat length–dependent expansion. However, the mechanism by which Polθ induces repeat expansions in a repeat length–dependent manner is unclear. Notably, DNA substrates used in this assay were gap-containing molecules, and the primer contained no repeat sequences and no hairpin. Thus, Polθ used an error-free primer to initiate DNA synthesis. When it fills a short repeat gap, for example, 15 or 20 repeats, Polθ may have sufficient processivity to quickly finish synthesizing the repeats and reach a nonrepeat template, which provides less of a chance for Polθ to make errors. When Polθ encounters a template with a large repeat number, such as 35 repeats, which is challenging for all DNA polymerases, it may have to pause a couple of times, and this may allow a repeat hairpin to form, leading to repeat expansion. Future studies are required to test these possibilities.

### Mn^2+^ stimulates Polθ's error-prone activity for repeat expansion

**Figure 4:**
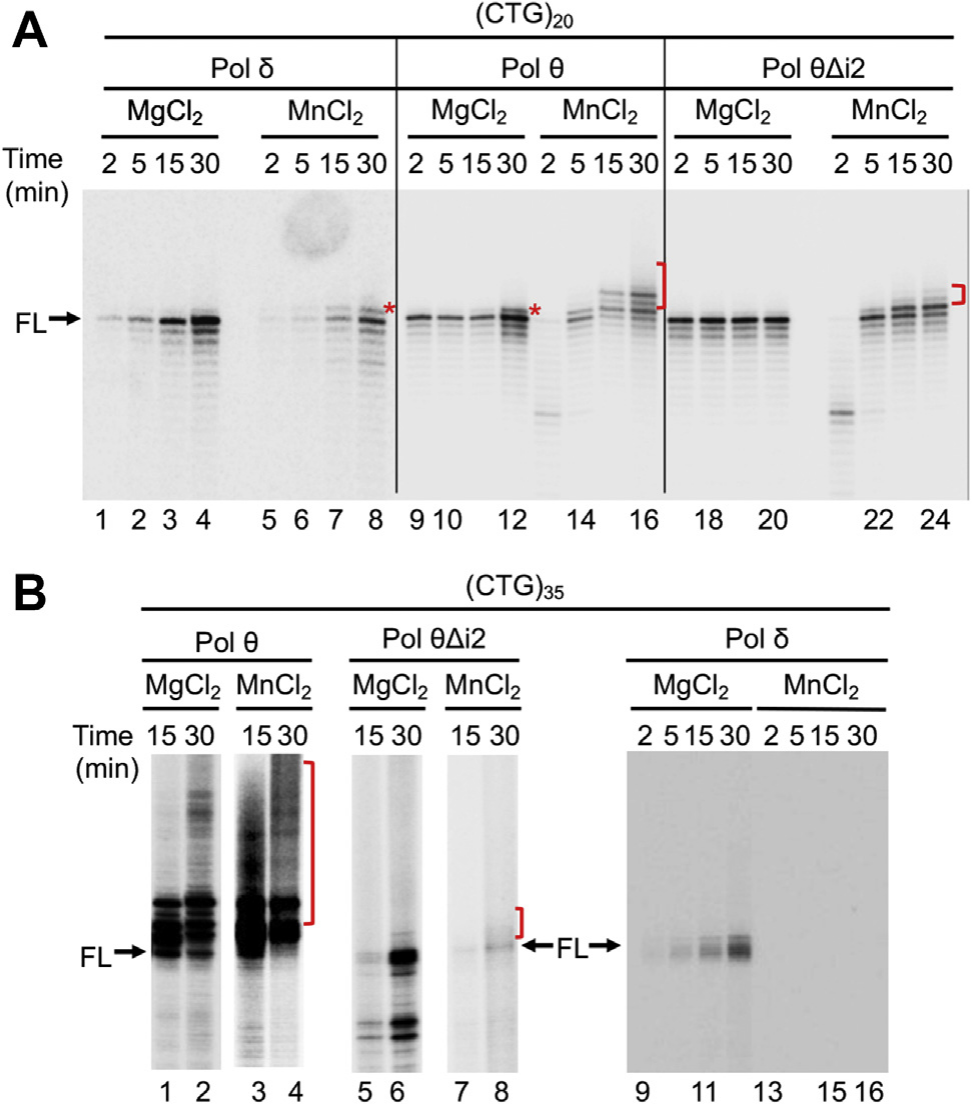
**Mn**^**2+**^**augments Polθ's repeat expansion capability.***A*, *in vitro* DNA synthesis assay using a synthesized oligonucleotide template containing 20 CTG repeats, which shows that Mn^2+^, but not Mg^2+^, triggers Polθ to conduct repeat expansions. *B*, *in vitro* DNA synthesis assay showing that Mn^2+^ stimulates Polθ, but not PolθΔi2 and Polδ, to carry out large repeat expansions when using an oligonucleotide containing 35 CTG repeats as a template. Polθ, DNA polymerase θ; PolθΔi2, insertion 2–depleted Polθ.

Divalent metal ion Mn^2+^ alters the structural flexibility of polymerase active sites in favor of error-prone synthesis (40, 41, 42, 43). To further determine whether the catalytic flexibility of the Polθ active site is responsible for the observed CAG/CTG repeat instability, we performed an *in vitro* DNA synthesis assay using oligonucleotide templates that contained 20 or 35 CTG repeats in the presence of Mg^2+^ or Mn^2+^. Although Polθ's TdT activity could only add one extra nucleotide when the (CTG)_20_ template was used for synthesis in the presence of Mg^2+^ (Fig. 4*A*, lane 12, red asterisk), it incorporated multiple nucleotides in the presence of Mn^2+^ (Fig. 4*A*, lanes 15 and 16, red bracket). We found that Mn^2+^ could also induce Polδ to generate a product that is one nucleotide (1-nt) bigger than the full-length product (Fig. 4*A*, lanes 7 and 8). We observed the same product in PolθΔi2-catalyzed reactions in the presence of Mn^2+^ (Fig. 4*A*, lanes 23 and 24). This product is probably derived from the 1-nt insertion during DNA synthesis, rather than from a TdT activity, particularly for reactions catalyzed by Polδ, which does not have a TdT activity. Strikingly, when the repeat length of the template was 35, Mn^2+^ further enhanced Polθ's error-prone activity, as we found more expanded products (Fig. 4*B*, red bracket) in reactions with Mn^2+^ than in those with Mg^2+^ (Fig. 4*B*, compare lane 1 with lane 3, and lane 2 with lane 4). These results indeed suggest that a more flexible active site induced by Mn^2+^ further enhances Polθ's error-prone capability. However, PolθΔi2 generated very few expanded products in the presence of Mn^2+^ (Fig. 4*B*, lanes 7 and 8), again suggesting that insertion 2 accounts for repeat expansion caused by Polθ.

We also noted that PolθΔi2 generated fewer expanded products in replicating a (CTG)_35_ template (Fig. 4*B*, lanes 7 and 8) than replicating a (CTG)_20_ template (Fig. 4*A*, lanes 23 and 24). We believe that this is related to the repeat length in the DNA template and the relatively high fidelity of PolθΔi2. Depleting insertion 2 from Polθ apparently converted an error-prone Polθ into a high-fidelity PolθΔi2, as judged by the fact that PolθΔi2-generated products are very similar to those produced by Polδ (Figs. 3 and 4). Our data presented here support the notion that polymerases with no insertion sequence in their catalytic domains, for example, Polδ and PolθΔi2, have difficulty synthesizing long triplet repeats, and that Mn^2+^ adds more difficulty for Polδ and PolθΔi2, but not for Polθ, in synthesizing long triplet repeats (Figs. 3 and 4*A*). To confirm this, we examined Polδ's ability to replicate the (CTG)_35_ template in the presence of Mg^2+^ or Mn^2+^. The results show that, although Polδ could generate a limited amount of full-length product in the presence of Mg^2+^ (Fig. 4*B*, lanes 9–12), it failed to produce any products in the presence of Mn^2+^ (Fig. 4*B*, lanes 13–16). Taken together, these results suggest that large repeat expansions can only be carried out by polymerases with a large insertion in their catalytic domain, such as Polθ, and that Mn^2+^ further enhances the error-prone property of Polθ, but not of polymerases with no insertion in their active sites, such as Polδ and PolθΔi2.

### Polθ is highly chromatin enriched in cell lines derived from patients with HD

**Figure 5:**
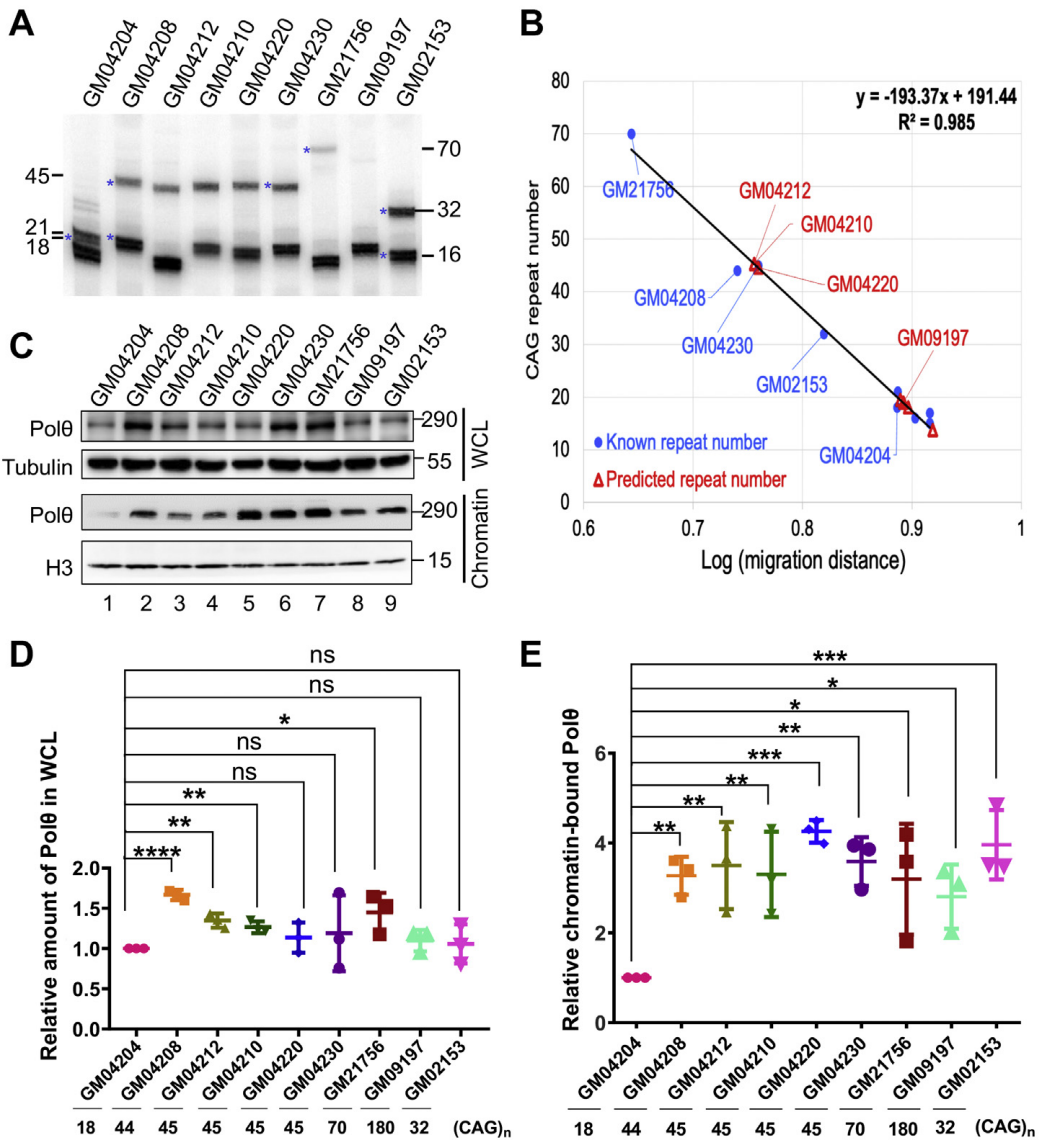
**Polθ's chromatin levels correlate with *HTT* CAG repeats.***A*, fractionation of the PCR products of *HTT* CAG repeat numbers of HD and non-HD cell lines by denaturing PAGE. PCR products were visualized by Southern blot analysis using a ^32^P-labeled (CTG)_5_ probe. *B*, determination of CAG repeat numbers by using a unique linear function (y = −193.37x + 191.44) between the CAG repeat number (y) and the logarithmic value of its migration distance. Cells lines with known or calculated CAG repeat numbers are shown in *blue* and *red*, respectively. *C*, determination of Polθ levels in whole-cell lysates (WCLs) and chromatin by Western blotting. *D* and *E*, quantification of Polθ levels in WCL (*D*) and chromatin (*E*) in HD and non-HD cell lines. ∗*p* < 0.05, ∗∗*p* < 0.01, and ∗∗∗*p* < 0.001. HD, Huntington’s disease; Polθ, DNA polymerase θ.

The data presented above strongly suggest that Polθ could be an important driving factor for CAG/CTG repeat expansion in HD. To explore this possibility, we set out to determine Polθ's expression and chromatin binding in cells derived from individuals with and without HD. We obtained seven fibroblast cell lines derived from patients with HD (GM04208, GM04212, GM04210, GM04220, GM04230, GM21756, and GM09197) and two non-HD cell lines (GM04204 and GM02153) from the Coriell Institute for Medical Research. The first five HD lines were from the same family, with GM04204 being an unaffected family member (44). To ensure the cell lines’ HD status, we amplified their *HTT* exon 1 sequence that contained CAG repeats by PCR and analyzed the resulting products in a polyacrylamide gel (Fig. 5*A*). We then calculated the CAG repeat numbers based on the migration distances of known CAG repeat numbers in HD cell lines, as the CAG repeat number (y) is a linear function of the logarithm of its migration distance (x) in gel electrophoresis: y = −193.37x + 191.44. The known CAG repeat numbers are 18/17 for GM04204, 45/19 for GM04230, 44/21 for GM04208, 70/15 for GM21756, 180/19 for GM09197, and 32/16 for GM02153 (Table S1). This analysis indeed confirmed the cell lines’ HD origin, with GM04212, GM04210, and GM04220 carrying an allele of at least 40 CAG repeats (Fig. 5*B* and Table S1). We noted that the 180-CAG repeat allele of GM09197 was not detectable in Figure 5*A*. This is probably because the large repeat number was hard to amplify, as only a small amount of the 70-CAG repeat product was obtained (Fig. 5*A*).

We then determined Polθ expression in these cell lines by Western blotting. In general, all cells expressed abundant Polθ (Fig. 5*C*, upper panel), but Polθ levels in whole-cell lysates (WCLs) in several HD cells were significantly higher than in non-HD control GM04204 (Fig. 5*D*). Strikingly, all HD cells exhibited a chromatin-bound Polθ level significantly higher than that in non-HD control GM04204 (Fig. 5*C*, lower panel, and Fig. 5*E*), which suggests that Polθ is efficiently recruited to chromatin in HD cells. For example, although GM04204 (Fig. 5*C*, lane 1) displayed an overall Polθ level slightly lower than that of GM04210 (Fig. 5*C*, lane 4) and GM04220 (Fig. 5*C*, lane 5), the former showed much less chromatin-bound Polθ than the latter ones (Fig. 5*E*). These results indicate a close association between Polθ chromatin binding and CAG repeat expansion. Interestingly, although GM02153 only carried an *HTT* with 32 CAG repeats, its chromatin-bound Polθ level is essentially the same as in several HD cell lines (Fig. 5*C*). This may explain why some patients display HD symptoms although their CAG repeat number is lower than the threshold 35 (45, 46). Thus, Polθ's chromatin level could be a reliable hallmark for HD diagnosis.

## Discussion

We show here that Polθ promotes CAG repeat instability in multiple ways. Polθ can extend a CAG repeat hairpin, which results in hairpin retention, and, in turn, CAG repeat expansion (Figs. 1 and 2). Polθ also induces large expansions when it copies a DNA template that contains 35 CAG repeats. This error-prone property of Polθ is due to its insertion 2, as Polθ△i2 cannot induce repeat expansions regardless of the primers or templates used (Fig. 3). Mn^2+^, which induces flexibility in polymerases’ active sites, further stimulates Polθ's error-prone activity (Fig. 4). Therefore, we have identified Polθ as a potent factor in promoting CAG repeat expansions in HD and other neurodegenerative diseases.

Polθ's involvement in CAG/CTG repeat expansion appears to be well justified. First, Polθ participates in BER (29), a DNA repair pathway implicated in CAG repeat instability (16). The brains of patients with HD are characterized by high levels of ROS (47), which can induce 8-oxoG adducts in guanine-rich CAG repeats (15). Repairing 8-oxoG requires the OGG1 glycosylase, which, together with the AP endonuclease, generates a small gap in the damaged DNA strand. This gap is then filled by Polθ or Polβ because both enzymes are required to repair oxidative damage (29). Because CAG repeats are prone to hairpin formation in the presence of a strand break/gap, a hairpin formed within the repeats can be utilized as a primer for DNA synthesis by Polθ (this study) or Polβ (23, 34), which leads to hairpin retention and large repeat expansion. In addition, Polθ possesses a large insertion 2 in the thumb domain (31), which creates a large flexible cavity in the DNA-binding surface (32, 33). This cavity facilitates misalignments between the primer and template during DNA synthesis (32), which leads to the addition of nucleotides to homopolymeric runs (30, 32). We showed that Polθ uses its large catalytic domain for CAG/CTG hairpin-priming synthesis (Figs. 1 and 2) and for large repeat expansions without a pre-existing hairpin structure in a repeat length–dependent manner (Fig. 3). Depleting insertion 2 makes Polθ a relatively high-fidelity polymerase, as PolθΔi2 no longer synthesizes error-containing DNA (Figs. 3 and 4). Finally, Polθ is highly chromatin-enriched in HD cells (Fig. 5). This gives Polθ the advantage of participating in DNA metabolic reactions, including the repair of oxidative DNA damage, so it can stimulate CAG repeat expansions in HD cells. However, how Polθ is preferentially recruited to chromatin in HD cells remains to be investigated.

Reports about whether people with an *HTT* gene that contains 27 to 35 CAG repeats are at risk for developing HD are controversial. Although CAG repeats in the 27 to 35 range are highly stable (48), some patients had the repeats extend into the HD onset range (>36), and some displayed HD symptoms although their CAG repeats remained unchanged (45, 46). This suggests that a portion of individuals with 27 to 35 intermediate CAG repeats can develop HD with or without the repeats expanding to ≥36. However, biomarkers that indicate HD onset and mechanisms that extend the repeats beyond the HD threshold are unknown. Our data presented here provide possible explanations for both questions. We showed that cells derived from patients with HD had significantly higher levels of Polθ in chromatin than cells from non-HD controls (Fig. 5). Surprisingly, we also found that, despite carrying an intermediate allele of 32 CAG repeats and being considered a non-HD control, the GM02153 cell line showed chromatin-bound Polθ levels similar to those of HD cells, rather than those of non-HD cells (Fig. 5). This suggests that the patient from whom GM02153 was derived might exhibit clinical features of HD. Furthermore, because Polθ carries out repeat length–dependent expansion, the polymerase can expand the intermediate (*i.e.*, 27–35) repeats to 36 and beyond. Therefore, Polθ concentration, particularly the chromatin-bound level, could be an important marker to determine the risk of HD for individuals with intermediate *HTT* alleles.

**Figure 6:**
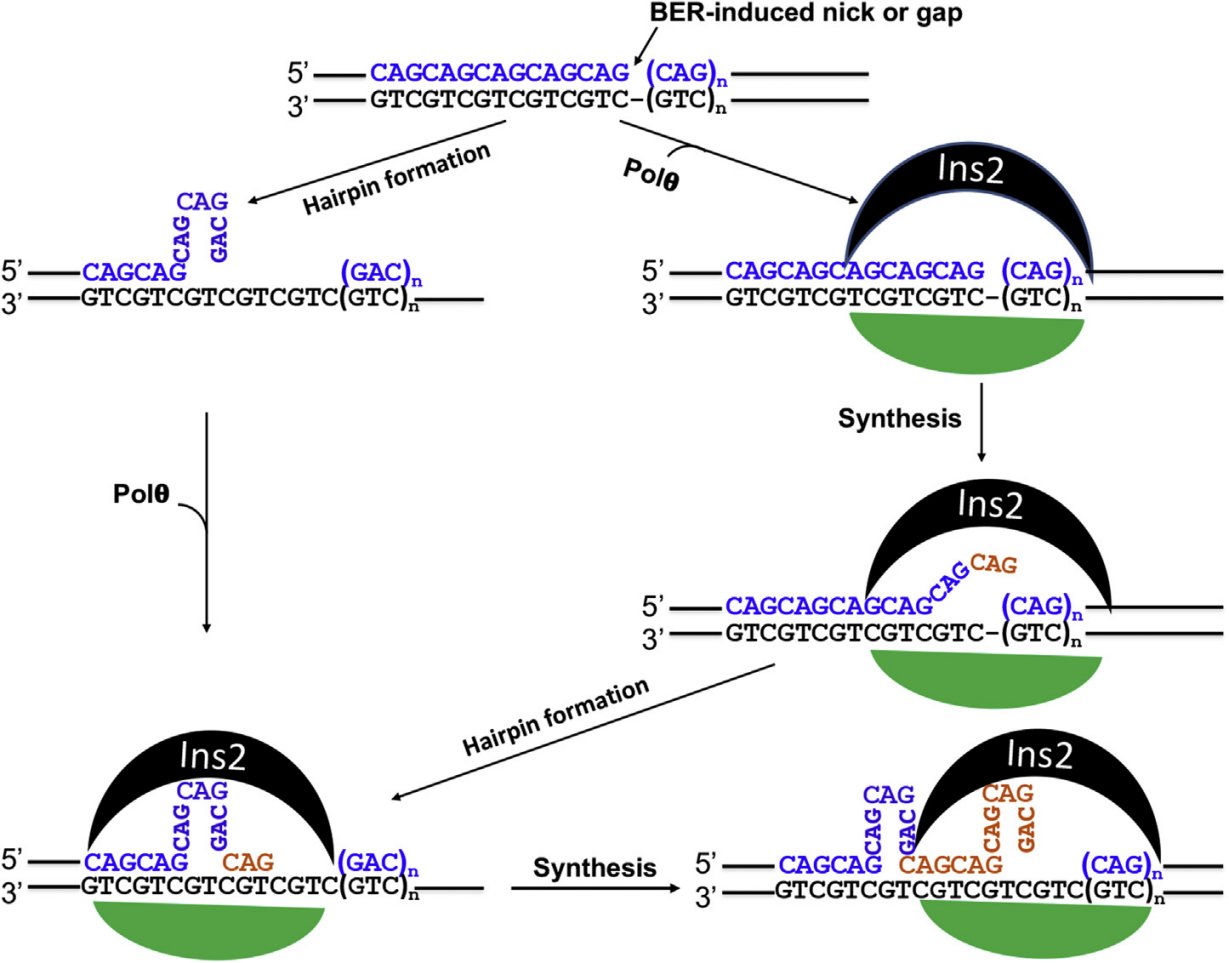
**Model of Polθ-catalyzed CAG repeat expansion.** OGG1-mediated repair of oxidative lesions within CAG repeats generates a strand break, which triggers the formation of a hairpin *via* strand slippage. Repair DNA synthesis is initiated when Polθ is recruited to the hairpin or nick. In the former case, Polθ utilizes the hairpin as a primer for extension, leading to hairpin retention and repeat expansion. In the latter case, Polθ incorporates nucleotides to the 3′ end of the nick. However, the newly incorporated nucleotides are trapped into the packet of insertion 2 (Ins2) to form a loop-out or a hairpin, which can grow into a big hairpin or shift out of insertion 2. The continuous synthesis by Polθ can trap a new hairpin formation in insertion 2, resulting in large expansion of the CAG repeats. OGG1, 8-oxo-guanine DNA glycosylase 1; Polθ, DNA polymerase θ.

Based on published data and the results presented here, we propose a model to elucidate the mechanism by which Polθ promotes CAG repeat expansions in nondividing neural cells during OGG1-mediated repair of 8-oxoG (Fig. 6). Once BER generates a strand break or a small gap within the CAG repeats, a CAG hairpin structure forms *via* strand slippage. Polθ can use the resulting hairpin as a primer for error-prone synthesis. Alternatively, Polθ incorporates nucleotides to the 3′ end of the nick/gap, and the newly incorporated nucleotides enter into the insertion 2–formed packet to form a loop-out, which can then easily convert into a (CAG)_n_ hairpin. The hairpin can either grow further or move out of the insertion 2 packet as the polymerase continues to synthesize. In the latter case, additional hairpins can form, which leads to variously expanded CAG repeats. However, the model, which is mainly based on our biochemical studies, requires thorough biological investigations in model organisms. For example, gene knockdown or KO of Polθ, Polβ, and/or other DNA polymerases may provide definitive evidence of how these polymerases promotes CAG repeat instability.

## Experimental procedures

### Cells and cell culture

Human fibroblast cell lines GM04204, GM04210, GM04230, GM04212, GM04208, GM04220, GM21756, GM09197, and GM02153 were purchased from the Coriell Institute for Medical Research and cultured in minimum essential medium with 15% fetal bovine serum (FBS). HeLa S3 cells were cultured in RPMI 1640 with 10% FBS. Human embryonic kidney (HEK) 293T cells were cultured in Dulbecco’s modified Eagle’s medium supplied with 10% FBS, and HEK 293GnTI^−^ cells were grown in suspension in FreeStyle 293 Expression Medium (Gibco) supplemented with 1% FBS. All human cell lines were cultivated at 37 °C in a humidified atmosphere containing 5% CO_2_.

### Nuclear extract and protein preparation

HeLa nuclear extracts were prepared as previously described (23). DNA sequences coding for human Polθ polymerase catalytic domain (Fig. 1*A*) and its insertion 2–deleted derivative PolθΔi2 were cloned into pLEXm and designated pLEXm-Polθ and pLEXm-PolθΔi2, respectively. pLEXm-PolθΔi2 was derived from pSUMO3-PolθΔi2 (a generous gift from Dr Richard T. Pomerantz, Temple University) by inserting the PolθΔi2-coding sequence into pLEXm after double digestions with AgeI and XhoI. Polθ and PolθΔi2 were expressed in HEK 293T cells and HEK 293GnTI^−^ cells, respectively, and purified as described previously (32). Polδ and RFC were expressed in High Five insect cells, and Polβ, replication protein A, and PCNA were expressed in *Escherichia coli*; all proteins were purified as previously described (49, 50).

### Hairpin primer extension assay

Unless otherwise mentioned, hairpin primer extension was assayed by Southern blot hybridization as described previously (23). Oligonucleotides that contained 15 CTG or 15 CAG repeats were annealed with ssDNA of M13mp18-(CAG)_10_ or M13mp18-(CTG)_10_ (51) to form CTG and a CAG hairpin substrate, respectively (Fig. 1*B*). Individual hairpin substrates were incubated with 30 μg HeLa nuclear extracts in the presence or absence of a polymerase (δ, θ, or θΔi2) for DNA synthesis at 37 °C for 30 min in a 40-μl reaction containing 110 mM KCl, 20 mM Tris-HCl, pH 7.6, 5 mM MgCl_2_, 1.5 mM ATP, 0.1 mM of various dNTP, and 0.05 mg/ml BSA. In the purified system, each reaction contained RFC (110 fmol) and PCNA (2 pmol) in addition to the indicated polymerase (0.1 μM). The resulting products were digested with BsrBI before electrophoresis through a 10% denaturing polyacrylamide gel, followed by Southern blotting using a ^32^P-labeled probe as described (23). The products were detected by an Amersham Typhoon phosphor imager.

### Preparation of WCL and chromatin-binding protein

Individual HD cells were harvested at 70% confluency and washed twice with ice-cold Dulbecco’s PBS. Chromatin fractions were prepared as described (52) and incubated with 1:1 (v/v) ice-cold 0.2 M HCl for 10 min on ice to denature and disassociate proteins from chromatin. The acidic solution was neutralized with 1:1 (v/v) of 1 M Tris•HCl (pH 8.0):1 U/μl Benzonase Nuclease (Sigma) and incubated on ice for 1 h to allow DNA digestions. The digested genomic DNA and insoluble proteins were removed by centrifugation. WCLs were prepared by resuspending cells in 1:1 (v/v) ice-cold 0.2 M HCl treated as chromatin fractions. Lysates were clarified by high-speed centrifugation (21,000*g*) for 15 min at 4 °C. Protein concentrations were determined by using the Bio-Rad Bradford Protein Assay reagent. After electrophoresis, chromatin-binding proteins and WCLs were analyzed for Polθ by Western blotting using a Polθ-specific antibody (Novus). Polθ bands were visualized and quantified by using a Bio-Rad ChemiDoc Imaging system.

### PCR amplification of CAG repeats and DNA sequencing analysis

PCR amplification of CAG repeats located in exon 1 of *HTT* in HD cell lines used forward (5′-ATGAAGGCCTTCGAGTCCCTCAAGT CCTTC-3′) and reverse (5′-CTGAGGCAGCAGCGG CTGTGCCTGCG-3′) primers. PCRs were performed in a volume of 25 μl containing 100 ng genomic DNA, 1.6 mM of each dNTP, 4 pmol of each primer, and 0.5 U Q5 DNA polymerase (New England Biolabs). After an initial denaturation of 4 min at 98 °C, 40 cycles of 45 s at 98 °C, 1 min at 68 °C, and 3 min at 72 °C were carried out, followed by a final extension of 10 min. PCR products were resolved in a 7.5% (wt/vol) denaturing polyacrylamide gel, followed by Southern blot analysis using a ^32^P-labeled (CTG)_5_ oligonucleotide probe. PCR products were then visualized by an Amersham Typhoon phosphor imager as described (49).

To analyze the expanded CTG repeats derived from Polθ-catalyzed primer extension, DNA bands (^32^P-labeled) were excised and eluted from gels, then PCR-reamplified for 35 cycles by REDTaq DNA polymerase (Sigma-Aldrich) using primers 5′-ACGTTGTAAAACGACGGCCA-3′ (forward) and 5′-CATGATTACGAATTC-3′ (reverse), essentially as described above. PCR products were cloned into a pGEM-T vector (Promega) and transfected into *E. coli* DH5-Alpha (Thermo Fisher). Plasmid DNAs were isolated and subjected to Sanger DNA sequencing (Source BioScience).

### Statistical analysis

All statistical assays and one-way ANOVA were performed by using GraphPad Prism 5.0 (GraphPad Software). Data were considered statistically significant if *p*-values were less than 0.05 or 0.001, as indicated.

## Data availability

All data are contained within the article.

## Supporting information

This article contains supporting information (53, 54, 55, 56, 57).

## Conflict of interest

All other authors declare that they have no conflicts of interest with the contents of this article.
