# Supplementary material for: DNA polymerase θ promotes CAG•CTG repeat expansions in Huntington’s disease via insertion sequences of its catalytic domain
Source: J Biol Chem. 2021 Aug 30;297(4):101144. doi: 10.1016/j.jbc.2021.101144 (PMC8463855; doi:10.1016/j.jbc.2021.101144)
Supplement: Supplemental Figures S1, S2 and Table S1 [file mmc1.docx]

Supporting information


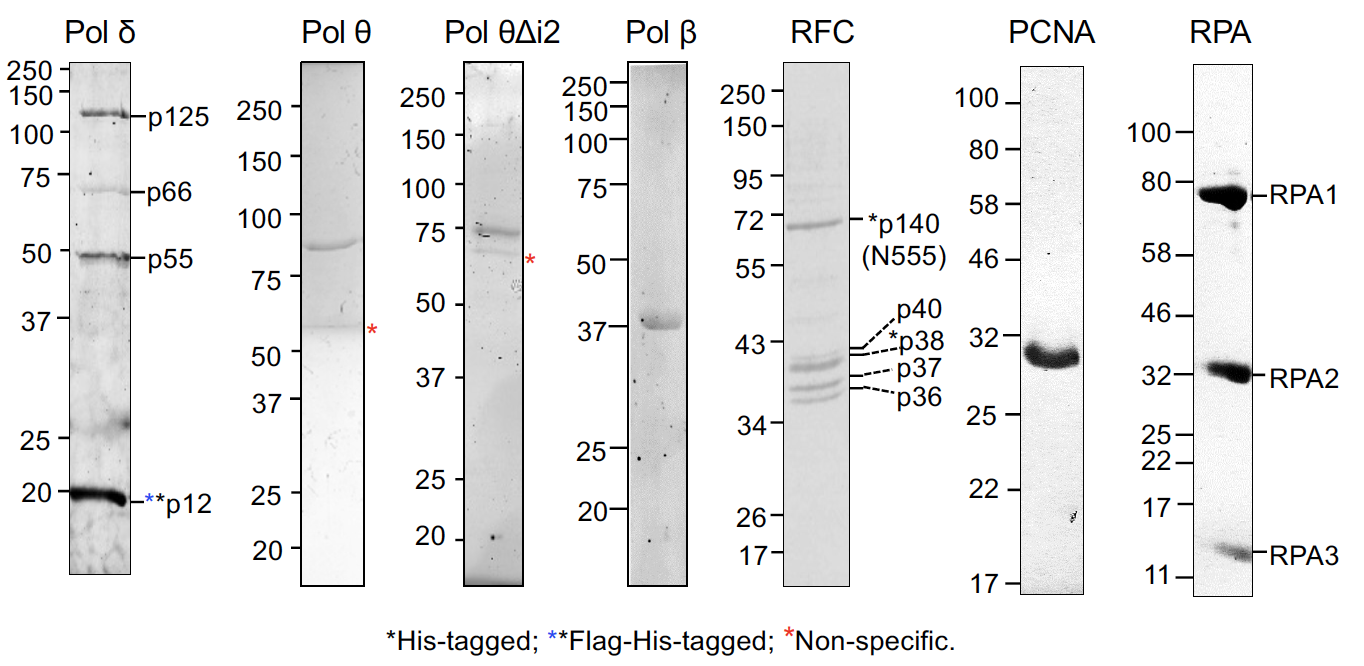


**Fig. S1.** Proteins used in this study. Polδ and RFC were expressed in insect cells through the baculovirus system; Polθ and PolθΔi2 were expressed in HEK93T cells; and Polβ, PCNA and RPA were expressed in *E. coli*. All proteins were purified to near homogeneity by column chromatography.

**
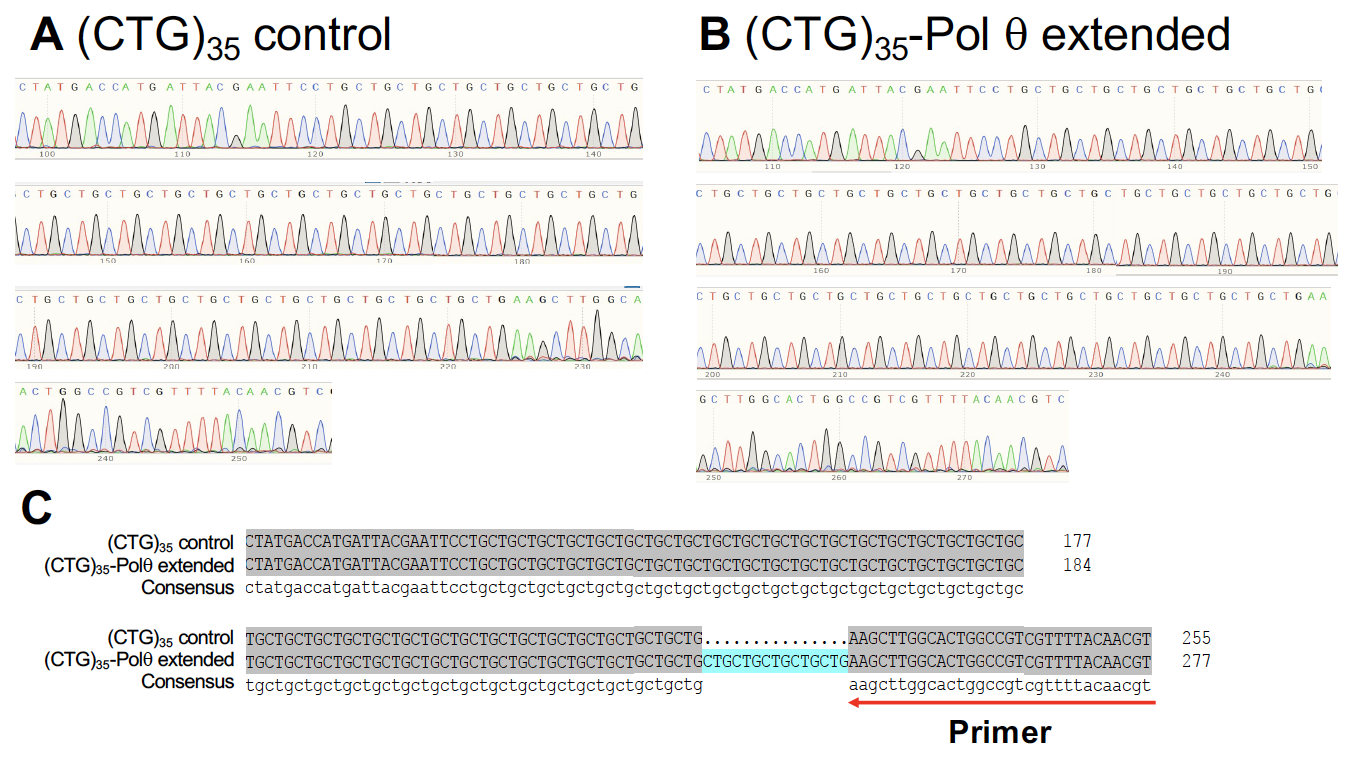
**

**Fig. S2.** DNA sequencing analysis of an extended product. DNA bands (full length and the expanded bands) were excised and eluted from gel, followed by PCR-reamplification for 35 cycles. PCR products were cloned into a pGEM-T vector and transfected into *E. coli* DH5-Alpha. Plasmid DNAs were isolated and subjected to Sanger DNA sequencing. (*A* and *B*) DNA sequences derived from PCR products of the full-length (CTG)_35_ control (*A*) and an expanded band (Fig. 3D) by Polθ (*B*). (C) Alignment of DNA sequences from *A* and *B*.

**Table S1.** Information on HD cell lines used in this study. Known and predicted CAG repeat numbers are indicated by black and red types, respectively.

| **Cell line** | **Gender** | ***HTT* Allele** | **Repeat number** | **HD Status** | **Reference** |
| --- | --- | --- | --- | --- | --- |
| GM04204 | M | 1 | 17 | No | (1) |
|  |  | 2 | 18 |  |  |
| GM04208 | M | 1 | 21 | Yes | (2) |
|  |  | 2 | 44 |  |  |
| GM04212 | F | 1 | 13 | Yes |  |
|  |  | 2 | 45 |  |  |
| GM04210 | M | 1 | 19 | Yes |  |
|  |  | 2 | 45 |  |  |
| GM04220 | F | 1 | 18 | Yes |  |
|  |  | 2 | 45 |  |  |
| GM04230 | F | 1 | 19 | Yes |  |
|  |  | 2 | 45 |  | (3) |
| GM21756 | F | 1 | 13 | Yes |  |
|  |  | 2 | 70 |  | (4) |
| GM09197 | M | 1 | 19 | Yes |  |
|  |  | 2 | 180 |  | (3) |
| GM02153 | F | 1 | 16 | No | (5) |
|  |  | 2 | 32 |  |  |

**References**

1. D. Cohen-Carmon *et al.*, Progerin-Induced Transcriptional Changes in Huntington’s Disease Human Pluripotent Stem Cell-Derived Neurons. *Molecular Neurobiology* **57**, 1768-1777 (2020).
2. M. de Mezer, M. Wojciechowska, M. Napierala, K. Sobczak, W. J. Krzyzosiak, Mutant CAG repeats of Huntingtin transcript fold into hairpins, form nuclear foci and are targets for RNA interference. *Nucleic acids research* **39**, 3852-3863 (2011).
3. M. B. Victor *et al.*, Striatal neurons directly converted from Huntington’s disease patient fibroblasts recapitulate age-associated disease phenotypes. *Nature Neuroscience* **21**, 341-352 (2018).
4. M. L. Ferlazzo *et al.*, Mutations of the Huntington’s Disease Protein Impact on the ATM-Dependent Signaling and Repair Pathways of the Radiation-Induced DNA Double-Strand Breaks: Corrective Effect of Statins and Bisphosphonates. *Molecular Neurobiology* **49**, 1200-1211 (2014).
5. P. A. Mollica ( 2015) DNA Repair Deficiency in Huntington's Disease Fibroblasts and Induced Pluripotent Stem Cells. in *Biological Sciences* (Old Dominion University, Old Dominion University Digital Commons), pp 1-172.
